# Titanium surfaces with biomimetic topography and copper incorporation to modulate behaviors of stem cells and oral bacteria

**DOI:** 10.3389/fbioe.2023.1223339

**Published:** 2023-07-10

**Authors:** Ruiying Li, Shuigen Li, Yi Zhang, Di Jin, Zhiming Lin, Xian Tao, Tianlai Chen, Liyuan Zheng, Zhisheng Zhang, Qianju Wu

**Affiliations:** ^1^ Stomatological Hospital of Xiamen Medical College, Xiamen Key Laboratory of Stomatological Disease Diagnosis and Treatment, Xiamen, Fujian, China; ^2^ Shanghai Ninth People’s Hospital, School of Medicine, Shanghai Jiao Tong University, Xiamen, China

**Keywords:** dental implants, copper, biocompatibility, antibacterial performance, osteogenesis

## Abstract

**Purpose:** Insufficient osseointegration and implant-associated infection are major factors in the failure of Ti-based implants, thus spurring scientists to develop multifunctional coatings that are better suited for clinical requirements. Here, a new biomimetic micro/nanoscale topography coating combined with antibacterial copper was simultaneously designed for Ti-based implant surfaces by adopting a hybrid approach combining plasma electrolytic oxidation and hydrothermal treatment.

**Results:** The biological interactions between this biofunctionalized material interface and stem cells promoted cellular adhesion and spreading during initial attachment and supported cellular proliferation for favorable biocompatibility. Bone marrow mesenchymal stem cells (BMMSCs) on the coating displayed enhanced cellular mineral deposition ability, higher alkaline phosphatase activity, and upregulated expression of osteogenic-related markers without the addition of osteoinductive chemical factors, which improved osseointegration. More interestingly, this new coating reduced the viability of oral pathogens (*Fusobacterium nucleatum* and *Porphyromonas gingivalis*)—the primary causes of implant-associated infections as indicated by damage of cellular structures and decreased population. This is the first study investigating the antibacterial property of dental implants modified by a hybrid approach against oral pathogens to better mimic the oral environment.

**Conclusion:** These findings suggest that biofunctionalization of the implant coating by surface modification methods and the incorporation of antibacterial copper (Cu) offer superior osteogenesis capability and effective antibacterial activity, respectively. These strategies have great value in orthopedic and dental implant applications.

## Introduction

Titanium (Ti) and its alloys have superior biocompatibility and desired mechanical properties in the field of clinical dental implantation (Haugen and Chen, 2022). Although Ti-based products occupy a large percentage of the dental market, limited osseointegration is a continuous challenge that restricts application of Ti-based dental implants. Peri-implantitis is an inflammatory reaction that is one issue in the inherent non-antibacterial characteristics of Ti-based dental implants. Another issue is the different degrees of loss in supporting bone surrounding the dental implant (Kloss et al, 2022). Hence, a new generation of orthopedic and dental implants with both antibacterial ability and osteoinductivity are desirable for better clinical outcomes. Thus, various modification approaches have been investigated (Mei et al, 2014; Bessa et al, 2022). Of these, plasma electrolytic oxidation (PEO) technology has attracted considerable attention recently due to its convenience and effectiveness. It is a relatively new technology in the field of Ti-based materials and can achieve favorable biological effects (Zhao et al, 2009; Al-Dulaijan and Balhaddad, 2022). Through PEO, a relatively rough and firmly adherent porous titania coating can be prepared on Ti-based implants. This coating contains anatase and rutile together with bioactive elements such as calcium (Ca) and phosphorus (P) originating from selective electrolytes. Numerous studies have demonstrated that a calcium/phosphate-rich titania coating fabricated by PEO could improve the biological performance of implants, thus making them potential candidates in dental applications (Krzakala et al, 2013; Zhou et al, 2013; Huang et al, 2022).

The oral environment is abundant with microorganisms and surgery can compromise host defenses and consequently facilitate bacterial attack (Zhang et al, 2013). PEO-modified dental implants offer inadequate antibacterial properties that restrict its application on a larger scale (Dias Corpa Tardelli et al, 2021). Hence, it is critical to equip dental implants with antibacterial properties to restrain initial bacterial adhesion and biofilm formation. Thus, strategies including the delivery or incorporation of antibacterial inorganic elements such as copper (Cu) or silver (Ag) into the surface of biomedical implants could be a simple and effective way to achieve this goal. In contrast to antibiotics, copper ions are non-specific bactericides that can act against a broad spectrum of bacterial species (Goudouri et al, 2014). They are essential trace elements in the human body, and studies have demonstrated that they play significant biological roles including the ability to enhance cell activity and proliferation of osteoblastic lineages and promote angiogenesis. These features make copper ions more biologically functional than silver ions (Ag^+^).

Hydrothermal treatment is an effective approach to loading inorganic ions and regulate surface chemical elements (Li et al, 2014). A hybrid approach that combines PEO and hydrothermal treatment to generate bioactive coatings on Ti-based implant surfaces is still in the early stages of development. Therefore, in the present work, we prepared a novel coating on Ti-based implant surfaces while simultaneously introducing the antibacterial copper by combining plasma electrolytic oxidation and hydrothermal treatment. Moreover, the interactions of this coating with oral microorganisms were assessed to address the design of Ti-based dental and orthopedic implants to better meet clinical requirements.

## Material and methods

### Abbreviation; day (d), hour (h), minute (min)

#### Fabrication of materials

**SCHEME 1:**
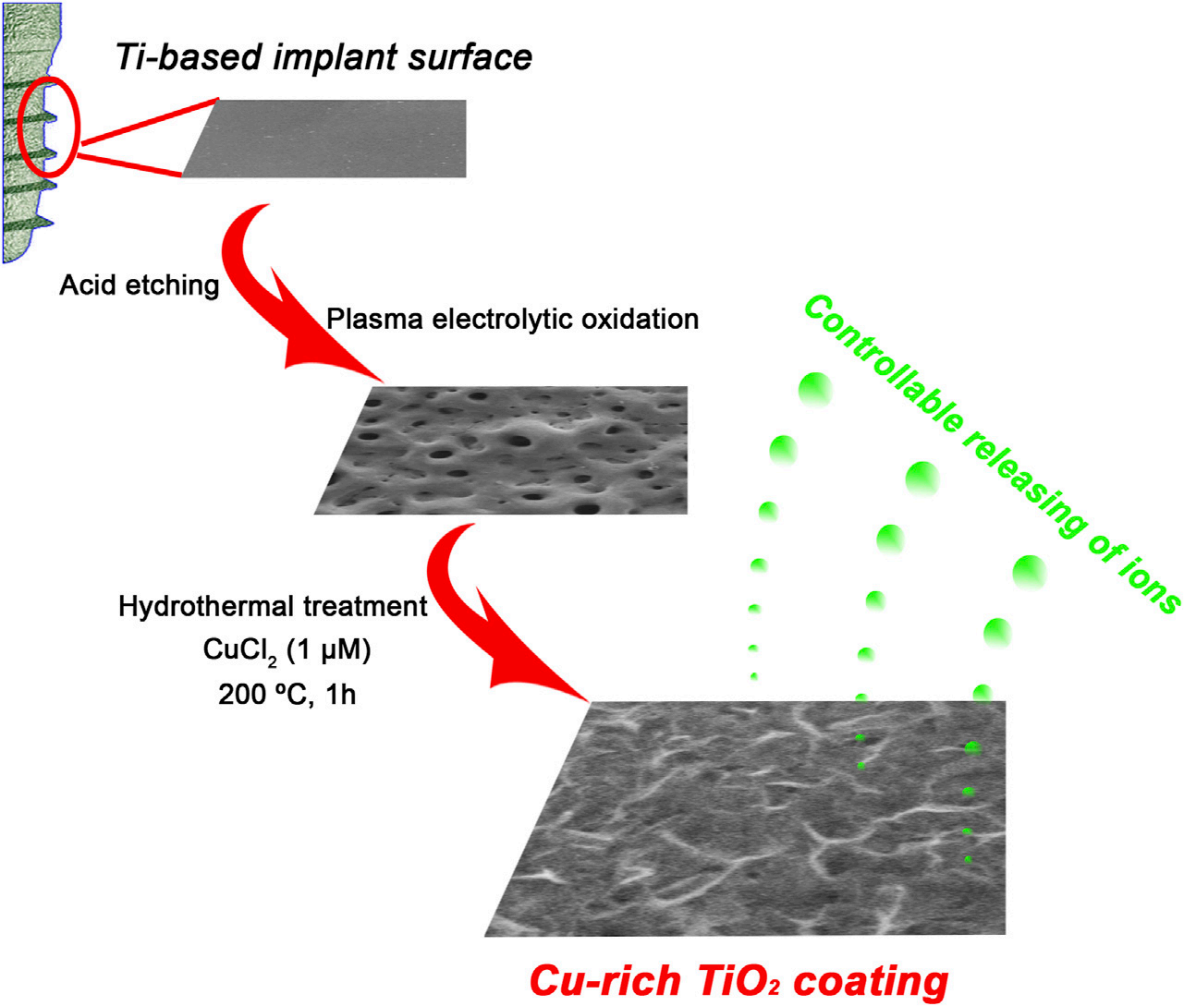
Schematic illustration for fabrication procedures of a bioactive Cu-TiO_2_ coating on Ti surface using plasma electrolytic oxidation (PEO) and hydrothermal treatment together.

Grade 1 pure Ti plates with purity of >99.85 wt% were cut into foils with dimensions of 1 × 1 × 0.1 cm^3^ or 2 × 2 × 0.1 cm^3^ for further use followed by acid cleaning in 5 wt% oxalic acid solution (100°C, 2 h) to obtain a homogeneous clean surface. A titania coating was then fabricated on a Ti surface via PEO in calcium/phosphate containing electrolyte, and the prepared samples were used as the control TiO_2_ group. In the experimental group, the hydrothermal method was employed to incorporate copper ions into the prepared TiO_2_ coating. It is indeed that the potential cytotoxicity of copper ions should be taken into consideration. As a consequence, a primary research on the role of copper ions with different concentrations for the biological activities of BMMSCs was performed in our previous work in order to optimize the experimental design (Wu et al, 2014). We found the concentrations of copper ranging from 1 nM to 1 μM turned out to show no significant cytotoxic effect for BMMSCs. Moreover, the concentration of 1 μM significantly upregulated the expressions of osteogenic genes of BMMSCs. On the basis of our foundation, 1 μM was chosen to modify the implant surface. Each PEO-pretreated Ti plate was placed in the Teflon-lined reaction vessel containing 1 μM CuCl_2_ aqueous solution for 1 h at 200°C. Afterward, the Ti plate was gently rinsed with ultrapure water and dried naturally. The final prepared specimen was named Cu-TiO_2_ (Scheme 1).

#### Surface characterization analysis

Field-emission scanning electron microscopy (FE-SEM; S-4800, HITACHI, Japan) was used to determine the surface topographies of samples, and the phase components were studied by an X-ray diffractometer (XRD; D/Max, Rigaku, Tokyo, Japan) fitted with a Cu Kα (λ = 1.541 Å) source ranging from 15° to 80° with a glancing angle of 1°. X-ray photoelectron spectroscopy (XPS; PHI 5802, Physical Electronics Inc., Eden Prairie, MN) with an Mg Kα (1,253.6 eV) source was used for the analysis of surface chemical compositions and states of different coatings.

#### Ion release detection

The samples of TiO_2_ and Cu-TiO_2_ were soaked in 10 mL Dulbecco’s Modified Eagle’s Medium (DMEM, Gibco, United States) and incubated at 37°C for 1, 4, 7, and 14 d. At each time point, the liquid extract was collected, and the concentrations of Ca/P/Cu ions released were tested by inductively coupled plasma mass spectrometry (ICP-MS; Nu Instruments, Wrexham, UK).(Yan et al, 2022).

#### Contact angle measurement

Contact angle measurements were performed to detect the surface wettability of samples under room temperature with atmospheric relative humidity of 30% by a contact angle instrument (Automatic Contact Angle Meter Model SL200B, Solon Information Technology Co., Ltd., China). The contact angles of five ultrapure water droplets were analyzed on each group repeatedly, and the results were expressed as mean ± SD.

#### Isolation and culture of bone marrow mesenchymal stem cells (BMMSCs)

BMMSCs derived from Wistar rats were isolated and cultured according to previously published procedures.(Wu et al, 2022). Briefly, both ends of the rat femurs were removed and the bone was then quickly rinsed out by Dulbecco’s Modified Eagle’s Medium (DMEM, Gibco, United States) with 10% fetal bovine serum (FBS, Gibco, United States) and 200 U/mL heparin (Sigma, United States). The primary cells were cultured under 5% CO_2_ and 37°C in DMEM supplemented with 10% FBS, 100 U/mL penicillin, and 100 U/mL streptomycin in an incubator. Non-adherent cells were removed, and the culture medium was renewed every 2-3 d. Cells were sub-cultured when they reached 80%–90% confluence, and passage 2–4 of BMMSCs were employed in the following *in vitro* experiments.

#### Cell adhesion and morphology observation

Cell counting was performed to detect the adhered cells on each sample at the initial seeding period of 4 h. Cells at the density of 4.0 × 10^4^ cells/well were seeded onto samples and incubated at 37°C for 4 h. The non-adhered cells were removed by PBS, and remaining cells were fixed in 4% paraformaldehyde for 30 min at 4°C and subsequently stained with DAPI (Sigma, United States) for 5 min at room temperature. The cell number was determined in five random fields at ×200 magnification of each sample with immunofluorescence microscopy (Olympus, Japan).

To detect the morphology of stem cells, samples were fixed and treated with 0.1% Triton X-100 and then blocked with 1% BSA for 30 min. Finally, the cytoskeletons were stained by incubating with Phalloidin-TRITC (Sigma, United States), and the cell nuclei were contrast-labeled by DAPI (Sigma, United States) visualized using immunofluorescence microscopy (Olympus, Japan).

#### Cell proliferation

The cell proliferation activity of the BMMSCs seeded on each sample was tested by MTT metabolic assay as previously described.(Wu et al, 2014). Initially, 2.0 × 10^4^ cells per mL were seeded onto each sample placed in 24-well plates for culture. At d 1, 4, and 8, the MTT solution (5 mg/mL) was added into the wells and incubated at 37°C for 4 h to form formazan, which was then dissolved in dimethyl sulfoxide (DMSO). Finally, the absorbance was measured at 490 nm with an ELX Ultra Microplate Reader (Bio-Tek, United States). The experiments were run in triplicate.

#### Osteogenic potential estimation

To investigate whether Cu-doped TiO_2_ coating could enhance the potential activity of osteogenic differentiation of BMMSCs seeded on implant surface, mineral deposition assay, alkaline phosphate (ALP) activity, and real-time quantitative polymerase chain reaction (RT-qPCR) assays were performed.

Cell mineral deposition assays were performed at d 14 by alizarin red S (ARS) assay. BMMSCs cultured on each sample were fixed in 95% alcohol for 15 min, immersed in 0.1% ARS solution (Sigma) for 30 min, and then washed with PBS three times before observation, moreover, the quantitative analysis was also carried out. For the ALP activity assay, BMMSCs cultured on each sample for 14 d were fixed with 4% paraformaldehyde and stained with an ALP kit (Beyotime, China). Quantitative ALP analysis was performed by measurement of optical density (OD) at 405 nm after incubation with p-nitrophenyl phosphate (pNPP) (Sigma, United States). The activity levels were normalized to total protein and presented as OD values at 405 nm per milligram of total protein.

For real-time quantitative polymerase chain reaction (RT-qPCR) assays, total RNA was extracted from BMMSCs seeded on each sample by using Trizol reagent (Invitrogen, United States). The real-time PCR operation was performed via a Bio-Rad real-time PCR system (Bio-Rad, United States), and the comparative ΔΔCt method was employed to calculate the relative expression of osteogenic genes such as osteocalcin (OCN), osteopontin (OPN), and bone morphogenetic protein-2 (BMP-2). The β-actin house-keeping gene was treated for normalization. The purified specific primers were synthesized commercially (Shengong, China) and the sequences are shown in Table 1. All experiments were run in triplicate.

**TABLE 1:** The sequences of specific primers for real-time PCR operation.

Gene	Prime sequence (F, forward; R, reverse)	Product size (bp)	Accession number
OCN	F: TCA​ACA​ATG​GAC​TTG​GAG​CCC	161	NM_013414.1
	R: GCA​ACA​CAT​GCC​CTA​AAC​GG		
OPN	F: CAA​GCG​TGG​AAA​CAC​ACA​GCC	165	NM_012881.2
	R: GGC​TTT​GGA​ACT​CGC​CTG​ACT​G		
BMP-2	F: ATG​GGT​TTG​TGG​TGG​AAG​TG	167	NM_017178.1
	R: TGT​TTG​TGG​AGT​GGA​TGT​C		
β-Actin	F: AGG​GAG​TGA​TGG​TTG​GAA​TG	107	NM_031004.2
	R: GAT​GAT​GCC​GTG​TTC​TAT​CG		

### Anti-oral pathogens assay

For the sake of better mimicking the oral environment, two selective bacteria, *Fusobacterium nucleatum* (Fn, ATCC25586, Gram-negative bacteria) and *Porphyromonas gingivalis* (Pg, ATCC33277, Gram-positive bacteria) were utilized to test the antimicrobial activity of each sample against oral pathogens. SEM were used to evaluate anti-oral pathogens ability of the prepared Cu-TiO_2_ coating. First, each sample was sterilized by 75 v/v% ethanol solution, and then a bacterial solution with the concentration of 10^7^ CFU/mL of Fn, was introduced onto the surface. After incubation under standard anaerobic conditions of 80% N_2_, 10% H_2_, and 10% CO_2_ at 37°C for 1 d, the samples were rinsed with phosphate buffered saline (PBS) three times, fixed in 3% glutaraldehyde, and dehydrated in a series of ethanol solutions (from 30 to 100 v/v%) for 10 min, followed by drying in hexamethyldisilizane (HMDS). Finally, the morphology of Fn on each sample were observed by SEM (SEM, S-3400, HITACHI, Japan).

For fluorescence staining, the viability of bacteria (Fn and Pg) on samples was investigated using a LIVE/DEAD BaclightTM Bacterial Viability Kit (Life Technologies, United States). The samples were incubated with the bacteria-containing medium (10^7^ CFU/mL) followed by rinsing with PBS. SYTO 9 and propidium iodide (PI) dyes were used to label the live and dead bacteria in green or red, respectively, for 15 min in darkness, and then the samples were examined with a confocal laser scanning microscope (CLSM, Leica, Germany).

### Statistical analysis

All statistical comparisons were measured via *t*-tests and analyzed by the SAS 8.2 software package (Cary, United States). The data acquired were represented as mean ± standard deviation (SD), and *p* < 0.01 was considered statistically significant and defined compared to the control TiO_2_ group.

## Results

### Materials characterization

**FIGURE 1:**
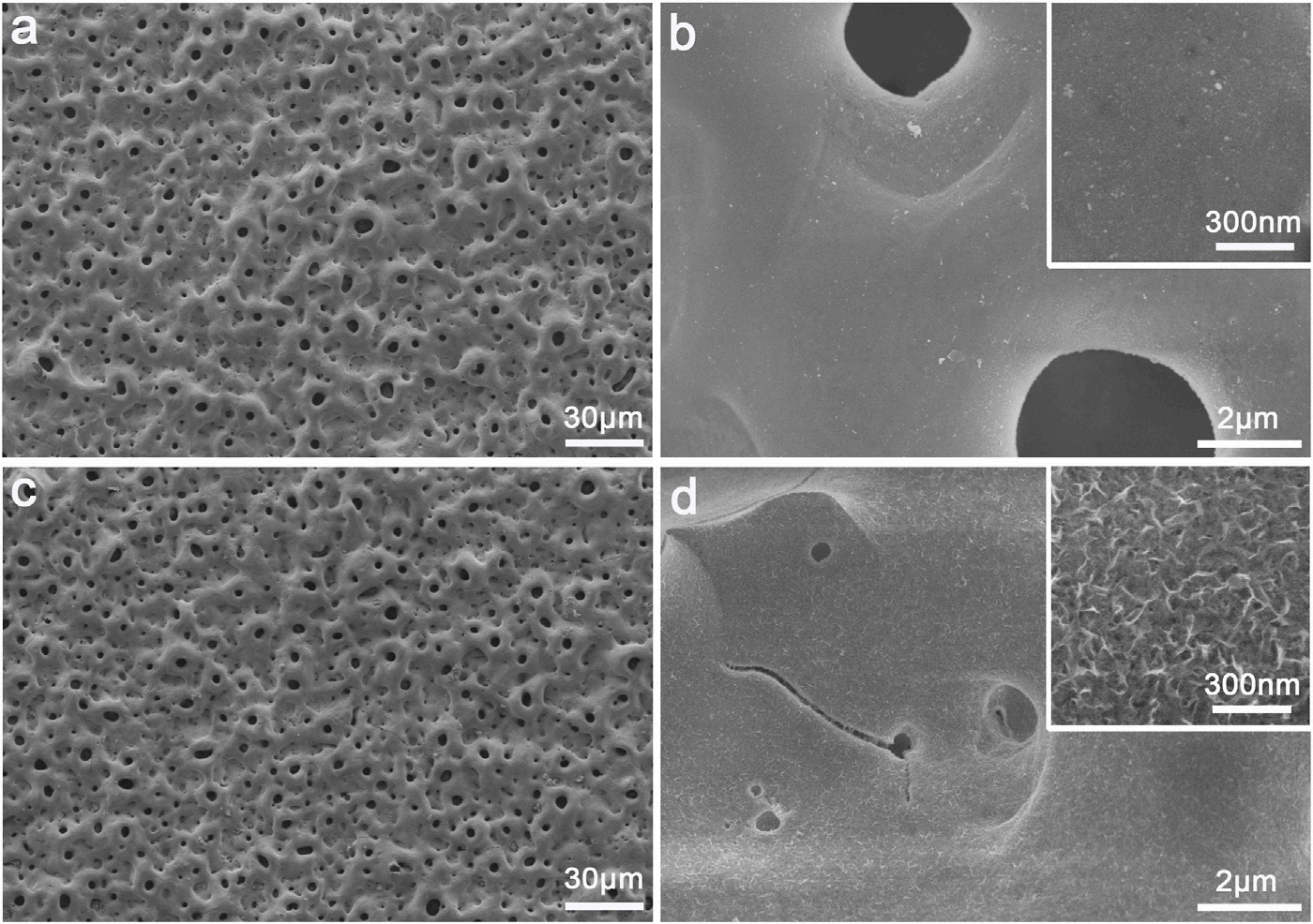
Surface morphology of the plasma electrolytic oxidized TiO_2_ coating **(A–B)** and Cu-TiO_2_ coating **(C–D)** observed by SEM at low and high magnifications together with the insets showing the corresponding higher-magnification pictures.

**FIGURE 2:**
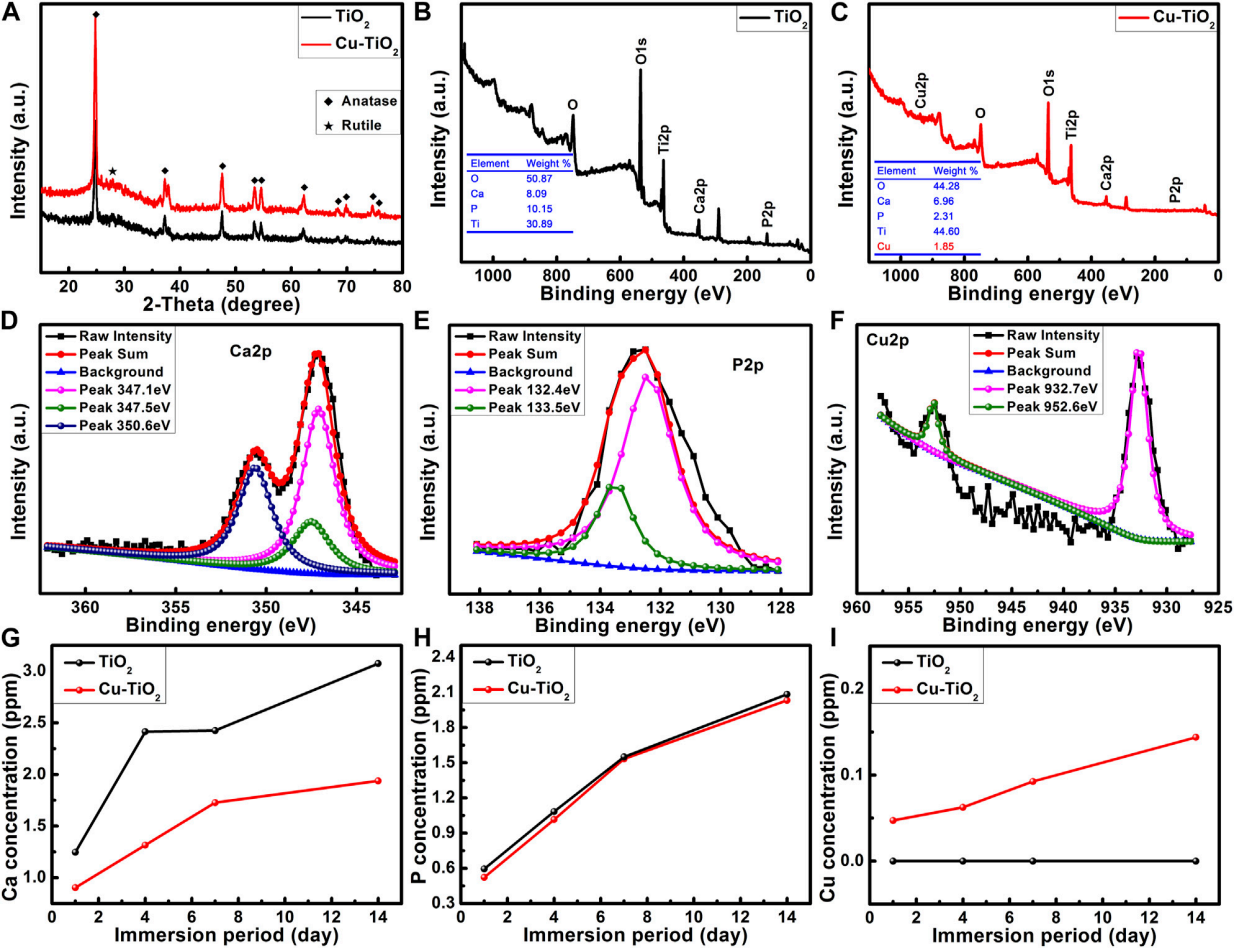
XRD patterns **(A)** and surface XPS full spectra **(B–C)** of samples TiO_2_ and Cu-TiO_2_ accompanied by the high-resolution XPS spectra of Ca 2p **(D)**, P 2p **(E)**, and Cu 2p **(F)** from sample Cu-TiO_2_ surface as well as the release profiles of Ca, P, and Cu ions within 14 d **(G–I)**.

The surface morphology of the PEO-treated and subsequent hydrothermally treated Ti plates was observed by FE-SEM. In Figure 1A, the surface of Ti after PEO treatment presented a rough and porous topography—there were numerous micron-sized holes distributed uniformly. The PEO-treated coating on Ti surface was mainly composed of anatase TiO_2_ with typical diffraction peaks (2θ = 25.2°, 38.0°, 48.1°, 53.8°, 62.8°, etc.), A small peak at 2θ = 27.4° could also be observed as the indicator of rutile phase according to the XRD (Figure 2A). After the hydrothermal treatment in copper-containing aqueous solution, the low magnification topography of the coating surface was not transformed significantly (Figure 1C) nor was the phase component; a unique nanopetal-like topography emerged on the TiO_2_ coating at high magnification (Figure 1D), whereas the PEO-treated Ti displayed a relatively smooth surface morphology (Figure 1B).

XPS analysis (Figures 2B, C) showed that oxygen (O), titanium (Ti), calcium (Ca) and phosphorus (P) could be detected from the PEO-treated Ti surface. Moreover, Cu with a content of 1.8 wt% was also detected from the sample treated with the hydrothermal treatment in CuCl_2_ aqueous solution; there were some losses of Ca and P during the hydrothermal treatment. Further high resolution XPS analysis was conducted for the Cu-TiO_2_ sample. Figure 2D shows that three peaks were consistent with the predominant ones at 347.1 eV and 350.6 eV corresponding to Ca 2p in Ca_3_ (PO_4_)_2_; another at 347.5 eV was assigned to Ca 2p in CaHPO_4_. P 2p (Figure 2E) had two peaks at 133.5 eV and 132.4 eV consistent with the P–O bonds in ${PO}_{4}^{3-}$ and ${HPO}_{4}^{2-}$ . The double peaks of Cu 2p located at 952.6 eV and 932.7 eV were attributed to the Cu 2p_1/2_ and Cu 2p_3/2_ in CuTiO_3_, respectively (Figure 2F). Figures 2G–I showed the release profiles of Ca, P, and Cu ions from each sample immersed in DMEM. Over 14 d, Ca and P ions were released from each sample in a sustained way. It existed statistic difference for the releasing amount of Ca ion between two groups, however, the amount of P ion for these two groups were similar without statistic difference. For Cu-TiO_2_ groups, the copper ions could also be released in a slow and sustained way, and at each time point, the releasing amount of Cu-TiO_2_ was remarkably higher than the TiO_2_ group.

### Surface wettability

**FIGURE 3:**
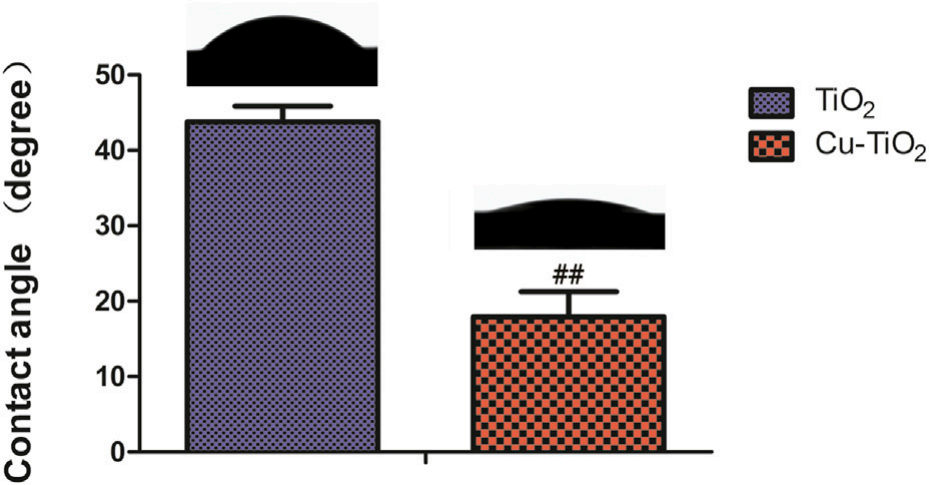
Contact angle measurement on different samples accompanied by corresponding typical ultrapure water droplet images. Notes: ##*p* < 0.01 vs. TiO_2_ group.

The water contact angles of samples TiO_2_ and Cu-TiO_2_ are displayed in Figure 3. The surface of Cu-TiO_2_ became more hydrophilic than that of TiO_2_ after the hydrothermal treatment, thus leading to a diminishing contact angle likely due to the generation of micro/nanostructured surface with copper incorporation. These surface changes altered the surface topography and chemical composition serving as two factors for material wettability. Moreover, it is widely accepted that hydrophilic surfaces can improve bioactivity and promote cell attachment, spreading, and proliferation.

### Cell adhesion, spreading and proliferation

**FIGURE 4:**
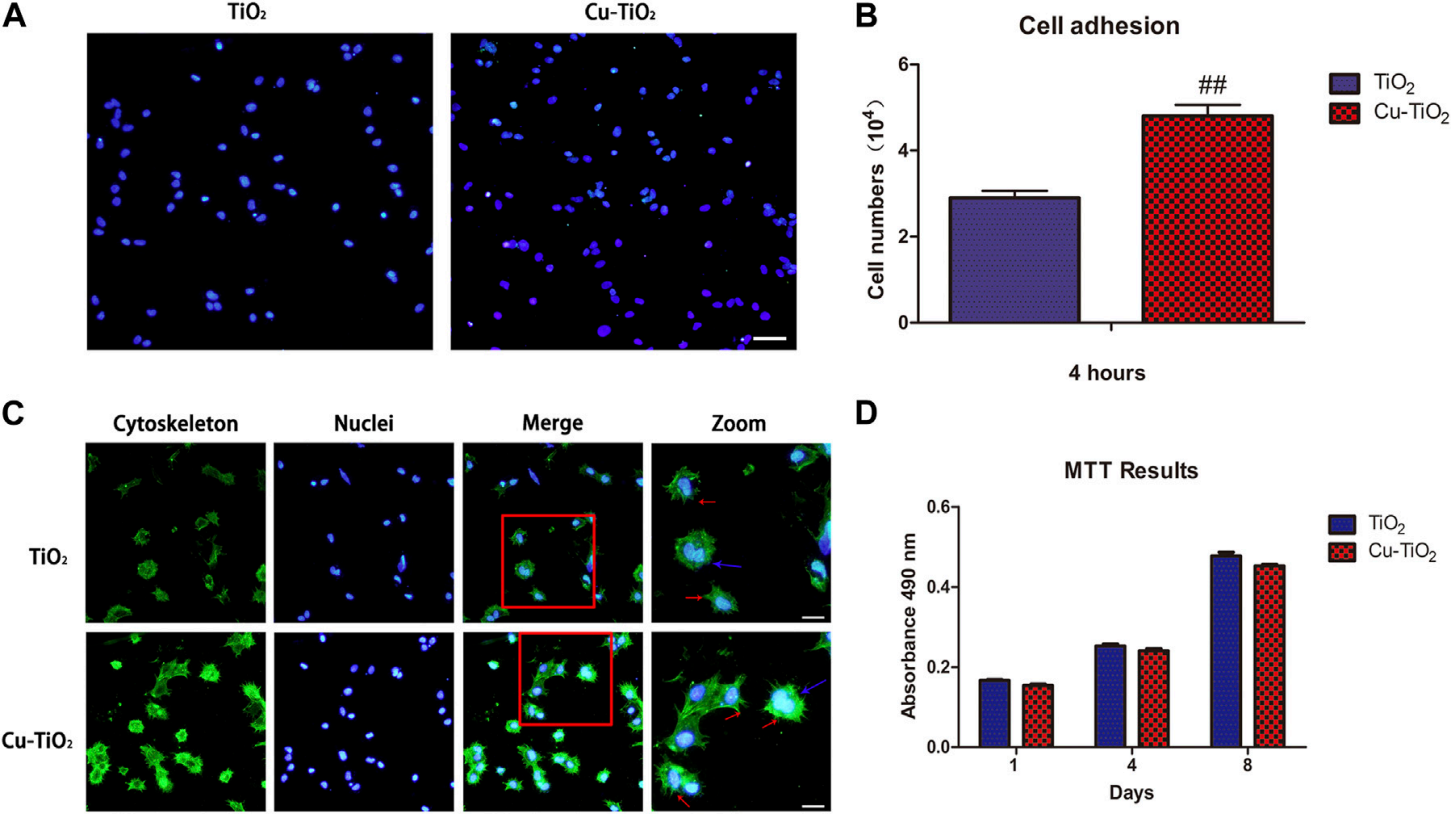
Cell initial adhesion, spreading, and proliferation ability evaluation. **(A)** Image of adherent cells with immunofluorescence microscopy (scale bar = 100 μm) together with the adhesion data **(B)**; **(C)** Immunofluorescence of cell spreading: The actin filaments (cytoskeleton) were labelled in green, and blue represented cell nuclei (scale bar = 100 μm); **(D)** MTT assay showed the proliferation ability of BMMSCs. All experiments were performed in triplicate. Notes: ## indicates *p* < 0.01 *versus* TiO_2_ group.

The biological responses of BMMSCs on two different coatings at the initial adhesion period were detected by immunofluorescence microscopy (Figures 4A–C). To investigate adhesion, cells attached onto both samples were detected after rinsing with PBS to remove non-adhered cells after 4 h of culture. The results reveal that the number of adherent cells on the Cu-TiO_2_ coating was significantly higher than the TiO_2_ coating control group. The quantitative data acquired by counting stained cellular nuclei suggests more adhesive cells on the Cu-TiO_2_ than TiO_2_ group with a statistically significant difference (*p* < 0.01, Figure 4B). This is consistent with Figure 4A.

Cytoskeletons were visualized by labelling to observe the cellular morphology of seeded BMMSCs (Figure 4C). The BMMSCs on sample TiO_2_ seemed to be round based on the lack of the noticeable filopodia extensions, while more pronounced filopodia extensions (indicated by red arrows) and extraordinary cellular elongation were apparent on the Cu-TiO_2_ surface under the same culture conditions. Mitosis phase cells (indicated by blue arrows) could be observed on each sample, thus suggesting that both surfaces were favorable for the initial adhesion and spreading of BMMSCs with no cytotoxicity.

The MTT assay was performed to determine the proliferation and vitality of BMMSCs cultured on the coatings (Figure 4D). Cell proliferation increased with time, and there was no significant difference between the two groups, demonstrating that the approaches of surface modification seen here did not adversely affect the viability of stem cells. The fabricated Cu-TiO_2_ implant coating possessed excellent biocompatibility.

### Osteogenic differentiation activity

**FIGURE 5:**
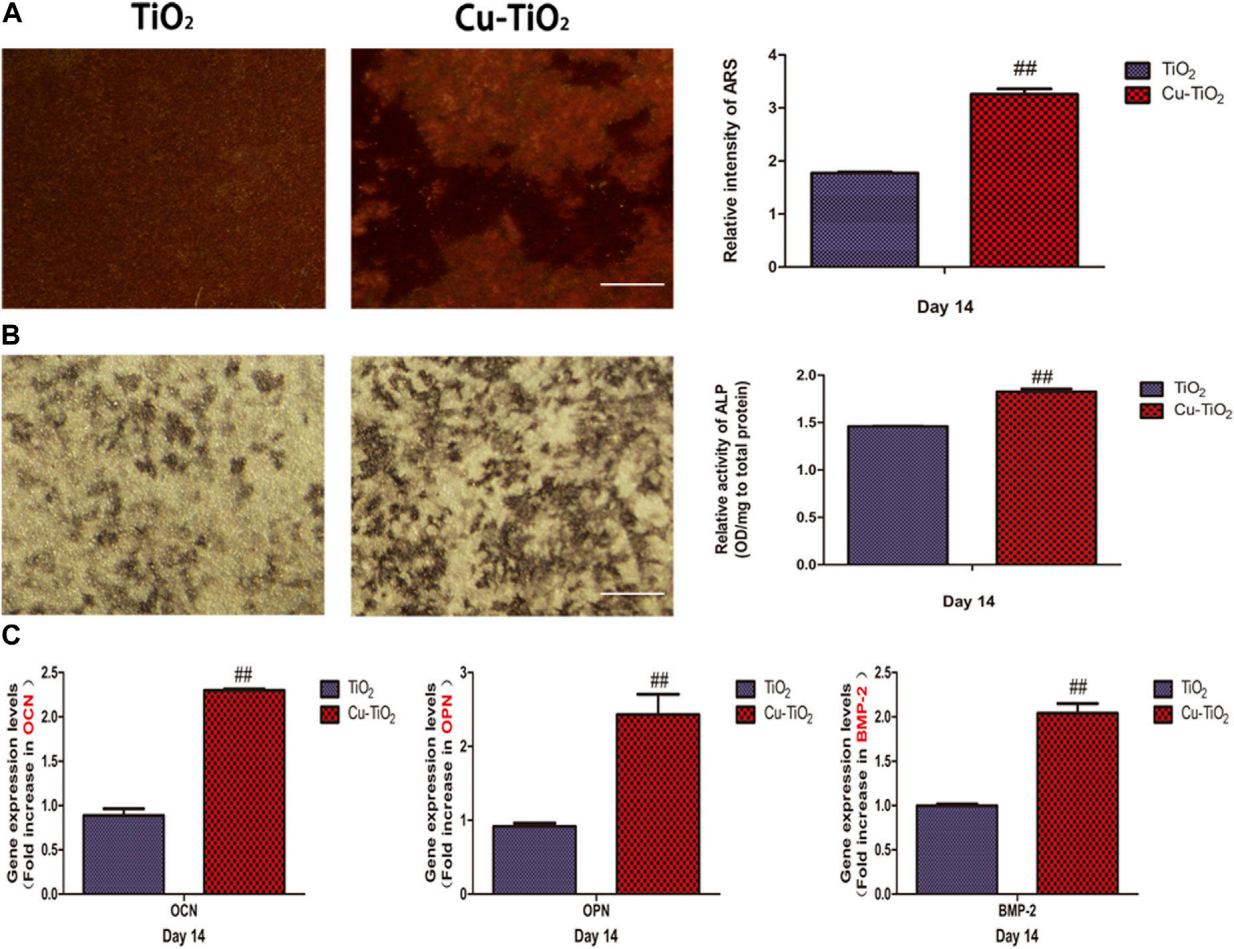
Mineral deposition assay **(A)** and alkaline phosphatase (ALP) activity **(B)** together with the expression levels of osteogenesis-related genes [**(C)**, OCN; OPN; BMP-2] for BMMSCs seeded on the TiO_2_ and Cu-TiO_2_ coating. All experiments were performed in triplicate. Notes: ## indicates *p* < 0.01 vs. TiO_2_ group. Scale bar = 75 μm.

Alizarin red S staining was conduct to determine the cell mineral deposition ability. As illustrated in pictures, Cu-TiO_2_ was higher than the other group, together with a statistic difference (Figure 5A). Additionally, ALP staining showed more extensive positive areas on the Cu-TiO_2_ sample, which was further verified by the quantitative analysis (Figure 5B). Furthermore, the RT-PCR analysis showed that the expression of selected osteogenic differentiation markers, including OCN, OPN, and BMP-2, were upregulated after 14 d of culture on the Cu-TiO_2_ coating (Figure 5C), thus indicating the promotion of osteogenetic differentiation potential of BMMSCs.

### Anti-oral pathogens assay

**FIGURE 6:**
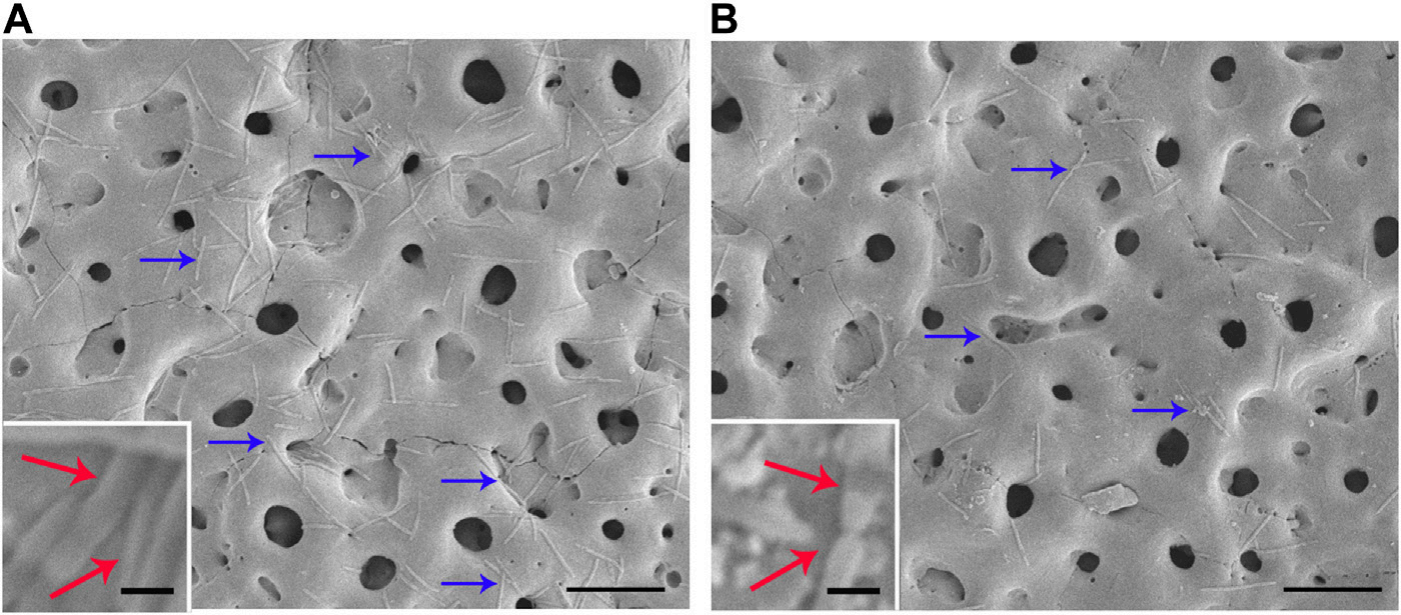
Typical photographs of Fn cultured on TiO_2_ coating **(A)** and Cu-TiO_2_ coating **(B)** by SEM. All experiments were performed in triplicate. Scale bar = 10 μm for the original pictures (right), and scale bar = 1 μm for the inset (left).

Fn and Pg were used to test the antimicrobial activity of each sample against oral pathogens. Figure 6 shows the typical morphology of Fn introduced on the two groups. Numerous bacteria were present on the surface of the TiO_2_ group (blue arrow in Figure 6A), and most of them displayed an intact cellular structure (red arrow in Figure 6A), thus suggesting strong biological activity. The amount of Fn on the Cu-TiO_2_ coating was reduced *versus* the former group. Damage to cellular structures was observed, which indicated that Fn was reduced in terms of survival on the Cu-TiO_2_ coating (as shown in Figure 6B).

**FIGURE 7:**
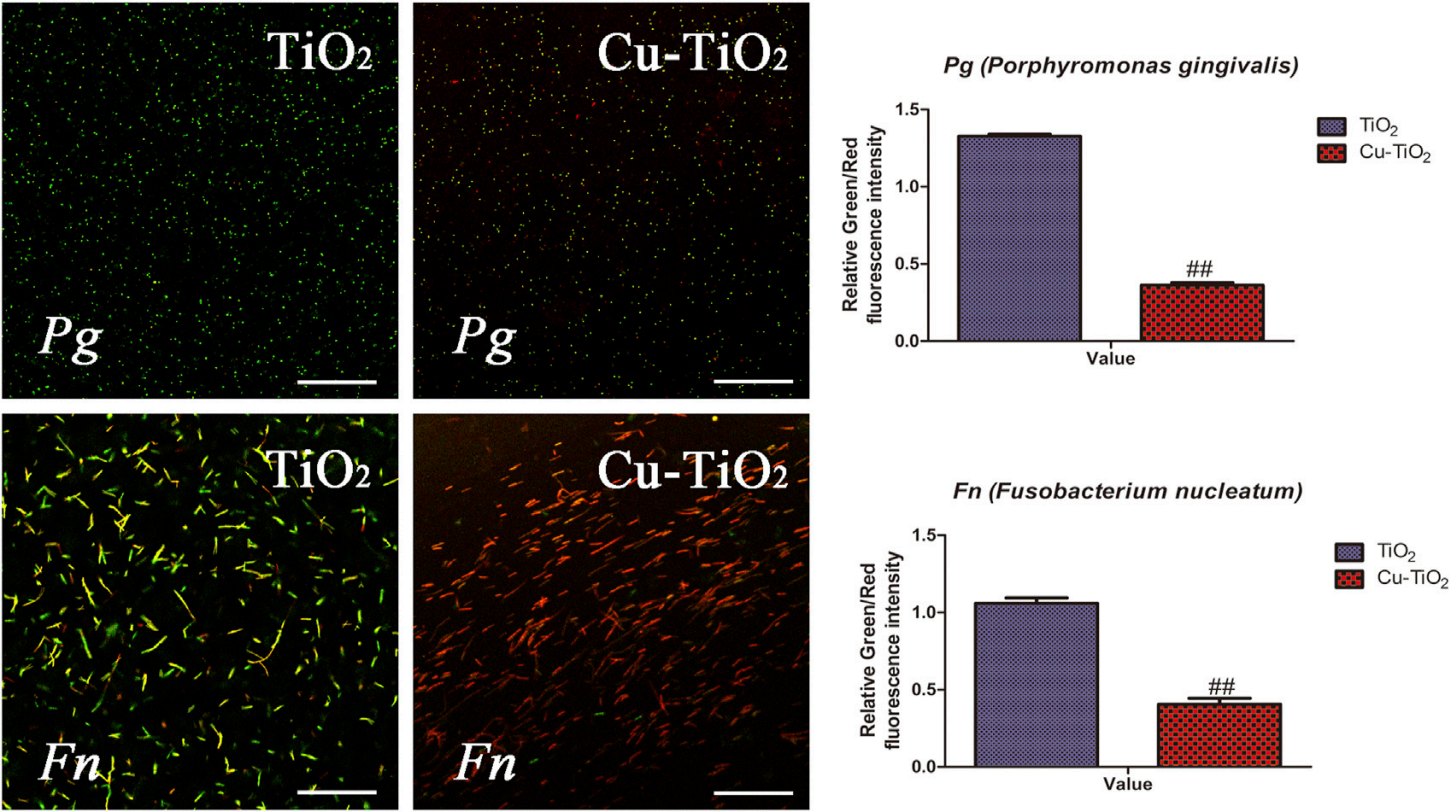
Typical confocal images of Fn (*F. nucleatum*, the upper row) and Pg (*P. gingivalis*, the bottom row) cultured on each sample for 1 d. The green fluorescence indicated live bacteria, and the dead ones are in red, together with the results of quantification. All experiments were performed in triplicate. Scale bar = 75 μm.

Figure 7 shows representative results of the live and dead assay for Fn and Pg after cultivation on the samples, respectively. The live bacteria with intact membranes were visualized in green fluorescence, and the dead ones with damaged membranes fluoresced in red due to the differences in spectral characteristics and the ability to penetrate healthy bacterial cells of SYTO 9 and PI. The amounts of dead bacteria of Fn and Pg were obviously pronounced while less vital ones were detected from the Cu-TiO_2_ coating than that on control group, especially for the population of Fn, showing its effective performance in killing the adhered oral bacteria.

## Discussion

A micron-sized structure was obtained on the Ti surface via the PEO process. This nanopetal-like topography was seen after the incorporation of copper ions by a hydrothermal method. More interestingly, the appearance of petal-like nanostructures is similar to the cross-section of tooth enamel after acid etching, i.e., the most highly mineralized tissue in the human body (Cassari et al, 2023; Wu et al, 2023). This result suggests that this nanometer topography plays a significant role in regulating the cells’ fate and micro/nanostructure from the biomimetic aspect and could possess excellent biological properties and enhanced stem cell functions (Wang et al, 2018; Bunz et al, 2022; Celesti et al, 2022). Our results showed that the Cu-TiO_2_ group with biomimetic micro/nanostructure was more hydrophilic. BMMSCs exhibit higher adhesion activity and more extensive cell spreading during the initial attachment period, and the trend of cell proliferation on two groups increased regardless of whether copper was incorporated or not during the culture period of 8 d, thus confirming excellent cytocompatibility of Cu-TiO_2_ coating. We further evaluated the osteogenic activity of stem cells on different samples and discovered that the mineral deposition ability and ALP activity of BMMSCs on Cu-TiO_2_ was improved. Moreover, the level of gene expression for selective osteogenic markers were all upregulated (OCN, OPN, and BMP-2), which were consistent with the results of ARS and ALP activity. Hence, this biofunctionalized surface is likely beneficial for guiding osteogenic differentiation of stem cells, which is critical for osseointegration (Li et al, 2023).

In the oral environment, more than 500 microbial strains play different roles in the oral biofilm formation and peri-implantitis (Brunetti et al, 2023; Ye et al, 2023). Many studies have demonstrated that there is a higher risk of pathogens colonization on the susceptible implant surface during the initial 6 h after surgery (Jin et al, 2019; Agarwalla et al, 2022), thus making the antimicrobial effects on the first day vital to clinical success. Hence, two representatives of oral pathogens (Gram-negative Fn and Gram-positive Pg), which are actively implicated in implant-associated infection, were immobilized onto the coatings for 1 d for antibacterial analysis. The Cu-TiO_2_ implant coating reduced the viability of bacterial species *versus* the control group, thus demonstrating its antibacterial properties. As a non-specific biocidal agent, copper can strongly destroy a broad spectrum of bacterial and fungal species (Hameed et al, 2022). The most accepted mechanism behind the antibacterial property is that copper could damage the activity of respiratory enzymes and destroy the integrity of bacterial membranes, thus disordering the biochemical processes leading to cell lysis or even cell death. However, the biological responses of cells to copper ions tend to show a concentration dependence on account of the potential cytotoxicity. The burst release and excessive doses of copper ions from implant’s surface are detrimental to cell function, which makes it critical to study this issue. Our time-releasing data indicate that the concentration of copper released accumulates to ∼0.15 ppm at 14 d, which is much less than the reported threshold of toxic concentration for human cells, thus making it possible to reduce the toxicity of copper and balance bacteria killing while supporting cell functions (Wu et al, 2023). Sufficient stem cell attachment and pronounced cell spreading could occupy more exposed space on the biomaterial surface against pathogens, thus helping to reduce the possibility of infection. To the best of our knowledge, there are no reports investigating the performance of implants incorporating antibacterial copper by this hybrid approach against oral pathogens. The approach described here is practical and valid, and can prevent implant-associated infections.

Biomaterials with hierarchical micro/nanotopographies can enhance the osteogenic activity of stem cells by imitating the natural hybrid structure of bone extracellular matrix (Santos-Coquillat et al, 2019; Sobolev et al, 2019; Vu et al, 2019). The proper amount of copper ions delivered in a controllable way of sustained release from the surface could induce some positive changes in the adjacent microenvironment and further regulate the biological responses of stem cells. Thus, the enhancement of cellular activities could be ascribed to the synergistic effect of the hierarchical surface topography and the bioactive copper element.

## Conclusion

A novel biomimetic micro/nanoscale topography coating incorporating antibacterial copper on a Ti-based implant surface was prepared via the hybrid approach combining PEO and hydrothermal treatment. The biological performance of this biofunctionalized implant surface could promote initial adhesion, proliferation, and further enhance osteogenic differentiation of stem cells. Its effectiveness in reducing the viability of oral pathogens such as Fn and Pg was demonstrated. Thus, the coating provides cytocompatibility, osteoinduction, and antibacterial properties. This study suggests that the biofunctionalization of implant coating and the incorporation of antibacterial copper could be achieved simultaneously to better meet clinical requirements with value in orthopedic and dental implant applications.

## Data Availability

The raw data supporting the conclusion of this article will be made available by the authors, without undue reservation.

## References

[B1] Agarwalla S. V. Ellepola K. Sorokin V. Ihsan M. Silikas N. Neto A. C. (2022). Antimicrobial-free graphene nanocoating decreases fungal yeast-to-hyphal switching and maturation of cross-kingdom biofilms containing clinical and antibiotic-resistant bacteria. Biomater. Biosyst. 8, 100069. 10.1016/j.bbiosy.2022.100069 36824379 PMC9934433

[B2] Al-Dulaijan Y. A. Balhaddad A. A. (2022). Prospects on tuning bioactive and antimicrobial denture base resin materials: A narrative review. Polym. (Basel) 15 (1), 54. 10.3390/polym15010054 PMC9823688 36616404

[B3] Bessa L. J. Botelho J. Machado V. Alves R. Mendes J. J. (2022). Managing oral health in the context of antimicrobial resistance. Int. J. Environ. Res. Public Health 19 (24), 16448. 10.3390/ijerph192416448 36554332 PMC9778414

[B4] Brunetti G. Valentini E. Berlutti F. Calvani P. Raponi F. Antonelli G. (2023). The effect of the electromagnetic field on metabolic-active bacterial biofilm experimentallyinduced on titanium dental implants. New Microbiol. 46 (2), 202–206. 37247241

[B5] Bunz O. Steegmann M. C. Benz K. Testrich H. Quade A. Naumova E. A. (2022). Human gingival fibroblast adhesion and proliferation on hydroxyapatite-coated zirconia abutment surfaces. Mater. (Basel) 15 (10), 3625. 10.3390/ma15103625 PMC9145355 35629651

[B6] Cassari L. Zamuner A. Messina G. M. L. Marsotto M. Chang H. C. Coward T. (2023). Strategies for the covalent anchoring of a BMP-2-mimetic peptide to PEEK surface for bone tissue engineering. Mater. (Basel) 16 (10), 3869. 10.3390/ma16103869 PMC10222618 37241496

[B7] Celesti C. Gervasi T. Cicero N. Giofre S. V. Espro C. Piperopoulos E. (2022). Titanium surface modification for implantable medical devices with anti-bacterial adhesion properties. Mater. (Basel) 15 (9), 3283. 10.3390/ma15093283 PMC9105612 35591617

[B8] Dias Corpa Tardelli J. Lima da Costa Valente M. Theodoro de Oliveira T. Candido Dos Reis A. (2021). Influence of chemical composition on cell viability on titanium surfaces: A systematic review. J. Prosthet. Dent. 125 (3), 421–425. 10.1016/j.prosdent.2020.02.001 32178882

[B9] Goudouri O. M. Kontonasaki E. Lohbauer U. Boccaccini A. R. (2014). Antibacterial properties of metal and metalloid ions in chronic periodontitis and peri-implantitis therapy. Acta Biomater. 10 (8), 3795–3810. 10.1016/j.actbio.2014.03.028 24704700

[B10] Hameed H. A. Hasan H. A. Luddin N. Husein A. Ariffin A. Alam M. K. (2022). Osteoblastic cell responses of copper nanoparticle coatings on Ti-6Al-7Nb alloy using electrophoretic deposition method. Biomed. Res. Int. 2022, 1–11. 10.1155/2022/3675703 PMC9042614 35496039

[B11] Haugen H. J. Chen H. (2022). Is there a better biomaterial for dental implants than titanium?-A review and meta-study analysis. J. Funct. Biomater. 13 (2), 46. 10.3390/jfb13020046 35645254 PMC9149859

[B12] Huang T. Wang H. Zhang Z. Feng K. Xiang L. (2022). Incorporation of inorganic elements onto titanium-based implant surfaces by one-step plasma electrolytic oxidation: An efficient method to enhance osteogenesis. Biomater. Sci. 10 (23), 6656–6674. 10.1039/d2bm00818a 36218838

[B13] Jin J. Fei D. Zhang Y. Wang Q. (2019). Functionalized titanium implant in regulating bacteria and cell response. Int. J. Nanomedicine 14, 1433–1450. 10.2147/IJN.S193176 30863070 PMC6390868

[B14] Kloss F. R. Kammerer P. W. Kloss-Brandstatter A. (2022). Risk factors for complications following staged alveolar ridge augmentation and dental implantation: A retrospective evaluation of 151 cases with allogeneic and 70 cases with autogenous bone blocks. J. Clin. Med. 12 (1), 6. 10.3390/jcm12010006 36614811 PMC9820942

[B15] Krzakala A. Kazek-Kesik A. Simka W. (2013). Application of plasma electrolytic oxidation to bioactive surface formation on titanium and its alloys. RSC Adv. 3 (43), 19725–19743. 10.1039/C3RA43465F

[B16] Li J. Zhang W. Qiao Y. Zhu H. Jiang X. Liu X. (2014). Chemically regulated bioactive ion delivery platform on a titanium surface for sustained controlled release. J. Mater. Chem. B 2 (3), 283–294. 10.1039/c3tb21102a 32261507

[B17] Li J. Zhao J. Xu Y. Xu A. He F. (2023). Titanium surface interacting with blood clot enhanced migration and osteogenic differentiation of bone marrow mesenchymal stem cells. Front. Bioeng. Biotechnol. 11, 1136406. 10.3389/fbioe.2023.1136406 37260826 PMC10227579

[B18] Mei S. Wang H. Wang W. Tong L. Pan H. Ruan C. (2014). Antibacterial effects and biocompatibility of titanium surfaces with graded silver incorporation in titania nanotubes. Biomaterials 35 (14), 4255–4265. 10.1016/j.biomaterials.2014.02.005 24565524

[B19] Santos-Coquillat A. Mohedano M. Martinez-Campos E. Arrabal R. Pardo A. Matykina E. (2019). Bioactive multi-elemental PEO-coatings on titanium for dental implant applications. Mater Sci. Eng. C Mater Biol. Appl. 97, 738–752. 10.1016/j.msec.2018.12.097 30678963

[B20] Sobolev A. Valkov A. Kossenko A. Wolicki I. Zinigrad M. Borodianskiy K. (2019). Bioactive coating on Ti alloy with high osseointegration and antibacterial Ag nanoparticles. ACS Appl. Mater Interfaces 11 (43), 39534–39544. 10.1021/acsami.9b13849 31590486

[B21] Vu A. A. Robertson S. F. Ke D. Bandyopadhyay A. Bose S. (2019). Mechanical and biological properties of ZnO, SiO(2), and Ag(2)O doped plasma sprayed hydroxyapatite coating for orthopaedic and dental applications. Acta Biomater. 92, 325–335. 10.1016/j.actbio.2019.05.020 31082568

[B22] Wang H. Zhang X. Wang H. Zhang J. Li J. Ruan C. (2018). Enhancing the osteogenic differentiation and rapid osseointegration of 3D printed Ti6Al4V implants via nano-topographic modification. J. Biomed. Nanotechnol. 14 (4), 707–715. 10.1166/jbn.2018.2551 31352944

[B23] Wu N. Gao H. Wang X. Pei X. (2023). Surface modification of titanium implants by metal ions and nanoparticles for biomedical application. ACS Biomater. Sci. Eng. 9, 2970–2990. 10.1021/acsbiomaterials.2c00722 37184344

[B24] Wu Q. Hu L. Yan R. Shi J. Gu H. Deng Y. (2022). Strontium-incorporated bioceramic scaffolds for enhanced osteoporosis bone regeneration. Bone Res. 10 (1), 55. 10.1038/s41413-022-00224-x 35999199 PMC9399250

[B25] Wu Q. J. Li J. H. Zhang W. J. Qian H. X. She W. J. Pan H. Y. (2014). Antibacterial property, angiogenic and osteogenic activity of Cu-incorporated TiO2 coating. J. Mater. Chem. B 2 (39), 6738–6748. 10.1039/c4tb00923a 32261870

[B26] Yan R. Li J. Wu Q. Zhang X. Hu L. Deng Y. (2022). Trace element-augmented titanium implant with targeted angiogenesis and enhanced osseointegration in osteoporotic rats. Front. Chem. 10, 839062. 10.3389/fchem.2022.839062 35273950 PMC8902677

[B27] Ye M. Liu W. Cheng S. Yan L. (2023). Efficacy of Adjunctive Chlorhexidine in non-surgical treatment of Peri-Implantitis/Peri-Implant Mucositis: An updated systematic review and meta-analysis. Pak J. Med. Sci. 39 (2), 595–604. 10.12669/pjms.39.2.7253 36950440 PMC10025710

[B28] Zhang W. Wang G. Liu Y. Zhao X. Zou D. Zhu C. (2013). The synergistic effect of hierarchical micro/nano-topography and bioactive ions for enhanced osseointegration. Biomaterials 34 (13), 3184–3195. 10.1016/j.biomaterials.2013.01.008 23380352

[B29] Zhao L. Chu P. K. Zhang Y. Wu Z. (2009). Antibacterial coatings on titanium implants. J. Biomed. Mater Res. B Appl. Biomater. 91 (1), 470–480. 10.1002/jbm.b.31463 19637369

[B30] Zhou J. Li B. Lu S. Zhang L. Han Y. (2013). Regulation of osteoblast proliferation and differentiation by interrod spacing of Sr-ha nanorods on microporous titania coatings. ACS Appl. Mater. Interfaces 5 (11), 5358–5365. 10.1021/am401339n 23668394

